# Acute Recovery after a Fatigue Protocol Using a Recovery Sports Legging: An Experimental Study

**DOI:** 10.3390/s23177634

**Published:** 2023-09-03

**Authors:** Gonçalo Silva, Márcio Goethel, Leandro Machado, Filipa Sousa, Mário Jorge Costa, Pedro Magalhães, Carlos Silva, Marta Midão, André Leite, Suse Couto, Ricardo Silva, João Paulo Vilas-Boas, Ricardo Jorge Fernandes

**Affiliations:** 1 Porto Biomechanics Laboratory (LABIOMEP-UP), University of Porto, 4200-450 Porto, Portugal; up201807261@fade.up.pt (G.S.);; 2 Faculty of Sport (CIFI2D), University of Porto, 4099-002 Porto, Portugal; 3 Tintex Textiles S.A., 4924-909 Viana do Castelo, Portugal; pedro.magalhaes@tintextextiles.com (P.M.); carlos.silva@tintextextiles.com (C.S.); 4 Centre of Nanotechnology and Smart Materials, 4760-034 Vila Nova de Famalicão, Portugal; 5 HATA Lda, 4740-183 Esposende, Portugal

**Keywords:** sports legging, localised heating, electrostimulation, compression, fatigue protocol

## Abstract

Enhancing recovery is a fundamental component of high-performance sports training since it enables practitioners to potentiate physical performance and minimise the risk of injuries. Using a new sports legging embedded with an intelligent system for electrostimulation, localised heating and compression (completely embodied into the textile structures), we aimed to analyse acute recovery following a fatigue protocol. Surface electromyography- and torque-related variables were recorded on eight recreational athletes. A fatigue protocol conducted in an isokinetic dynamometer allowed us to examine isometric torque and consequent post-exercise acute recovery after using the sports legging. Regarding peak torque, no differences were found between post-fatigue and post-recovery assessments in any variable; however, pre-fatigue registered a 16% greater peak torque when compared with post-fatigue for localised heating and compression recovery methods. Our data are supported by recent meta-analyses indicating that individual recovery methods, such as localised heating, electrostimulation and compression, are not effective to recover from a fatiguing exercise. In fact, none of the recovery methods available through the sports legging tested was effective in acutely recovering the torque values produced isometrically.

## 1. Introduction

Recovery and fatigue are essential components of physical performance and are closely related. Recovery is the process by which the body restores its physiological and psychological state after a period of stress or exertion [1], whereas fatigue is the temporary inability to maintain a desired level of physical or mental performance [2]. The recovery process can be influenced by numerous factors (such as nutrition, hydration and sleep [3]) and can be enhanced by using strategies such as active recovery, massage and cold/hot water immersion [4]. It is important that the community engaged in sports performance understand the importance of recovery and fatigue management to enhance coaches, medical personnel and practitioners’ performance and minimise the risk of injuries.

Besides the basics of recovery, techniques such as electrostimulation, localised heating and compression can also be used as effective recovery tools [5,6]. Electrostimulation is a sequence of stimuli that is provided by electrodes put superficially on the skin [7] and entails applying a series of intermittent stimuli to the superficial skeletal muscles to activate the intramuscular nerve branches and cause visible muscle contractions [8]. The possible effects of electrostimulation are enhanced blood flow, speeding up the removal of muscle metabolites [9] and the promotion of recovery by reducing muscle pain through central and peripheral mechanisms [7]; however, adequate intensities must be used as excessive intensities might lead to partial ischemia and insufficient ones may not increase blood flow. The literature still presents some controversy on this matter, with studies showing that electrical stimulation is not as effective as is supposed to be; this is mostly because the protocols applied are very heterogeneous and this fact must be taken into account [10].

Heating therapy is frequently prescribed for the treatment of musculoskeletal injuries or to protect the muscle from potential damage [11]; nonetheless, heat therapy has been proposed as a viable therapeutic strategy to control the progression of exercise-induced muscle damage according to the same authors. The underlying mechanisms that justify the application of such therapy are the elevated muscle tissue temperatures that can upregulate heat shock proteins and other signalling molecules [12] and some studies have shown that heat treatment given either before or after eccentric contractions results in an increased recovery for muscular strength, muscle endurance and soreness measures [13,14]. Localised heating may increase muscle function, endurance and strength and reduce soreness [15], as well as having benefits for cardiovascular health [16] and improvements in quality of life [17]; however, the ways in which this intervention helps in recovery following exercise-related muscle damage is still unknown [15].

The use of external compression is known to affect a few cellular and hemodynamic processes [18]. The application of a mechanical pressure to the body compresses the tissues underneath, reducing the space available for swelling [19] and consequently lessening the inflammatory response [20]. The use of compression garments in the recovery from exercise-induced muscle damage has been the subject of some controversy, with some authors supporting their use [21] and other authors reporting no additional benefit related with the use of compression garments [22]; however, no adverse effects are expected with the use of compression garments concerning recovery from damaging exercise [22,23,24].

Promoting adaptation to training loads, lowering the risk of injury and enhancing the body’s capacity to repeat high-level physical performances requires strategies to maximise recovery following exercise training or competition [25]. Innovative technologies in which a single device may hold different recovery techniques already exists [5] but, notwithstanding the potential benefits of combining recovery methods, there is still a need for determining the efficacy of using multiple strategies concurrently. The aim of the current study was to fill this research gap by incorporating electrostimulation, localised heating and compression in a single garment to be used in the practitioners’ lower limbs and analysing its efficacy on acute recovery. We hypothesised that this legging can promote acute recovery by combining different methods and muscle groups. This is the first study that has used a sports legging that assembles three recovery methods and analyses its efficacy on acute fatigue recovery.

## 2. Materials and Methods

### 2.1. Participants

Eight subjects (six male and two female, 28.4 ± 4.6 years old) who were tier 1 recreationally active, i.e., subjects who meet World Health Organization’s minimum activity guidelines (according to the Participant Classification Framework [26]), participated in the current study. Their main physical characteristics were 72.1 ± 13.1 kg body mass, 175.1 ± 8.4 cm height and 24.0 ± 3.7 body mass index. Participants were excluded if they fulfilled one of the following criteria: age not between 18 and 35 years, musculoskeletal injury within the six months prior to the evaluation or reported experiencing any form of discomfort during the experiments. Once a participant consented to enrol in this study, a dialogue with research staff took place to confirm his/her eligibility. Data were collected by the same team of evaluators and the volunteers were asked to cease intense or moderate exercise 24 h before the data collection and to not drink any energetic beverages prior to exercise. Ethical approval was obtained from the local ethical committee (CEFADE 14 2023).

### 2.2. Experimental Design

**Figure 1:**
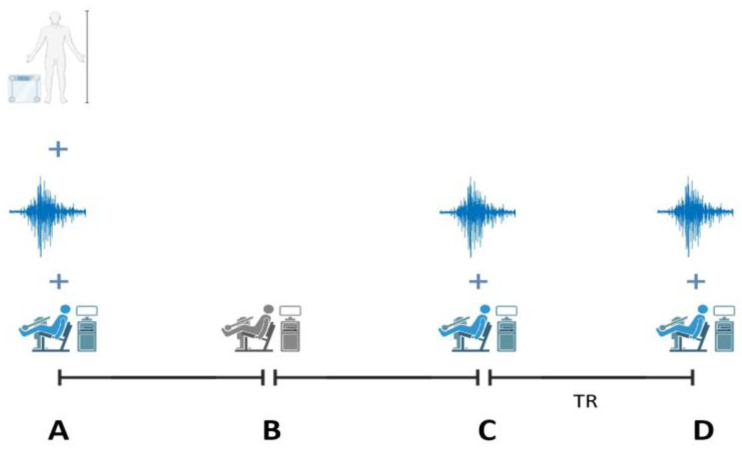
Schematic representation of the assessment and fatigue induction protocol with anthropometric evaluation and maximum voluntary isometric contractions (MVICs) (**A**) fatigue induction protocol (**B**), MVICs (**C**) MVICs and (**D**) MVICs + recovery treatment (TR).

To assess the fatigue recovery effect, subjects were immediately evaluated in three fixed instants for each selected method (Figure 1), i.e., before and after the fatigue induction protocol and 5 min after the recovery process. All subjects underwent an anthropometric characterisation consisting of height and body mass measurements. Dynamometric evaluations of maximum voluntary isometric contractions were performed using the Biodex© System 4 (Biodex Multi-Joint System 4, Biodex Medical System, Shirley, NY, USA) isokinetic dynamometer, allowing us to obtain peak torque as the principal outcome. This apparatus was also used to perform the fatigue protocol consisting of coupled eccentric and concentric isokinetic knee extension and flexion and ankle plantar flexion contractions repeated until the individual was unable to reach 70% of the concentric peak torque value achieved in the first 10 repetitions. The fatigue protocol duration was dependent on the individual initial average peak torque and a tendency was seen when subjects with higher peak torque values had a diminished capacity to endure the fatigue protocol in time. The mean and standard deviation of contractions for the control group were 72.25 ± 9.22, electrostimulation 69.13 ± 8.61, compression 92 ± 13.96 and localised heating 59.25 ± 8.99. Immediately after the fatigue protocol, subjects performed a set of five maximum voluntary isometric contractions for further comparisons, followed by the specific recovery treatment.

Electromyographic (EMG) activity was recorded throughout the experiment via electrodes placed directly on the skin following the recommendations of SENIAM [27,28]. Participants’ skin was shaved and 70° alcohol was applied to the stimulation sites. For evaluating the quadriceps, the vastus lateralis was selected and two electrodes were placed at two-thirds distance on the line from the anterior iliac spine superior to the lateral side of the patella in the direction of the muscle fibres, 20 mm apart. The biceps femoris electrodes were placed at 50% distance on the line between the ischial tuberosity and the lateral epicondyle of the tibia, oriented in the direction of the line between the ischial tuberosity and the lateral epicondyle of the tibia. For the gastrocnemius lateralis, electrodes were placed at one-third distance on the line between the head of the fibula and the heel, oriented in the direction of the line between the head of the fibula and the heel (see [29] for a detailed description of the methodological aspects regarding surface EMG assessment).

### 2.3. Intervention

**Figure 2:**
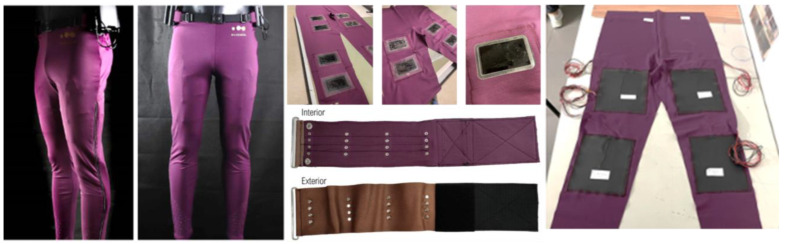
Left panel, middle upper and lower and right panels represent the sports legging global solution and individual solutions for electrostimulation, compression/massaging and localised heating, respectively.

Three different recovery protocols were applied using the sports legging (Figure 2); however, it is important to state that these methods do not work simultaneously but alternatively. Using emerging technologies associated with materials and processes, namely the integration and printing of electronic devices, this project’s main goal was to develop textile solutions with appealing designs conceived for the treatment of muscular fatigue. A strong connection to technology and different lines of investigation associated with engineering were expected at the beginning of this project. The sports legging integrates three recovery methods, each directed towards a specific muscle, i.e., electrostimulation, compression/massaging and localised heating directed towards the quadriceps, gastrocnemius and hamstrings, respectively (Figure 2). The project also included the development of a mobile application, together with the communication and hardware system as well as a wireless power supply system, thus keeping the system functional autonomously to provide the customer with full service and ease of use.

Participants had all recovery treatments (electrostimulation, localised heating and compression) for the interventions over the course of three distinct days, with at least 24 h between each treatment to allow for recovery. For all protocols, excluding the control intervention, subjects showed up at the lab and engaged in a series of concentric contractions for the muscle group that would be assessed that day, i.e., quadriceps, hamstrings and gastrocnemius. After the warm-up and before the pre-fatigue evaluation, a series of coupled eccentric–concentric contractions were carried out to familiarise the participants with the fatigue induction protocol. On a different day, the individuals in the control condition completed the same warm-up, which comprised a set of concentric knee extension contractions, and then proceeded to the protocol illustrated in Figure 1. The control group underwent a passive recovery treatment, which is the only distinction between the three recovery treatments and this group. The participants were previously accustomed to using the Biodex System 4 (Biodex Multi-Joint System 4, Biodex Medical System, Shirley, NY, USA) for the collection of torque data.

#### 2.3.1. Localised Heating

The localised heating involved the connection of wires to an electronic control belt debiting 10 W per heating zone, fixing the tension at 12 V. Different connexions were tested and, ultimately, we opted to choose the one that presented the least electric resistance, normalise the heat distribution in the heating zones. The maximum heating capacity was established at 60 °C. For the heating of the hamstrings, we followed a 20 min protocol at a 43.3 ± 2.8° mean temperature [30]. We chose this protocol because the heating protocols available that we found mainly involved whole-body exposure and our sports legging can only heat the hamstrings. From this point, we decided to standardise the duration of all protocols to 20 min.

#### 2.3.2. Compression

To improve the performance of the compression bands, the sports legging was instrumented with shape memory alloys of the bands because of the excellent thermomechanical behaviour [31] and shape memory polymers, and the implementation of a seam was adopted to confine the springs, crossing the entire length of the band to guarantee its stability and safety. The final version of the massage and compression sleeve consumed an electrical current of 1 A and operated at a voltage of 12 V. To be able to execute the 20 min compressing/massaging protocol, we had to tighten the compression sleeves around the gastrocnemius (Figure 2) and the pre-compression performed prior to the start of the protocol determined the pressure in the recovery process. We selected a 20 min protocol and standardised that to all recovery protocols based on data from the literature and the opinion of two physiotherapists [32].

#### 2.3.3. Electrostimulation

For the electrostimulation, leggings with two layers of mesh were created, with the electrodes sewed onto the inner layer to be in direct contact with the user and no solutions applied to the outer layer to standardise the appearance of the solution and hide all the connections. Layers of neoprene were used to improve the contact with the skin and all the connections are linked to a belt with magnetic connectors.

To carry out the electrostimulation protocol, we had to determine the individual intensity at which we had visible but not painful muscle contractions. We compared stimuli from sports leggings and a DS8R Biphasic Constant Current Stimulator (Digitimer^®^ DS8R, Welwyn Garden City, UK) with the same features as the legging (Table 1). First, the participants were dressed in the leggings, different intensities were tested and, based on the subject’s feedback, we selected a comfortable intensity. After this procedure, we went compared it with the Digitimer DS8R to see if there were any differences between them. This procedure was performed to normalise the intensities between subjects. EMG signals were acquired using MP100 BIOPAC Systems Inc., Goleta, CA, USA, and AcqKnowledge (version 3.9.1.6, BIOPAC Systems Inc., Goleta, CA, USA, If the EMG activity did not increase, the minimum intensity for maximum EMG activity was established. A possible reason for the recorded values of both systems being different is since the Digitimer^®^ apparatus calculates the impedance and modulates the voltage to maintain the current intensity.

#### 2.3.4. Control Group

After participants had gone through all the interventions, they were assigned to a control condition where they performed knee extensions. The participants passed through the control group 48 h after the last protocol they performed to ensure adequate rest. For further analysis, we recorded torque and EMG data for pre- and post-fatigue and post-recovery moments. For the recovery protocol, we instructed the subjects to lie down on a chair and recover for the next 20 min.

### 2.4. Data Analysis

Data related to the maximum isometric torque were acquired concomitantly with EMG values through an analogue input in the MP100 BIOPAC Systems Inc. (USA) with a 1000 Hz sampling frequency. Dynamometry and EMG data were filtered and processed through MATLAB environment routines (version R2022a, MathWorks©, Natick, MA, USA). Dynamometry data went through a 4th-order recursive lowpass Butterworth filter with a 6 Hz cut-off frequency. EMG data were previously filtered with a 4th-order recursive bandpass filter with 20 and 450 Hz cut-off frequencies; then, a full-wave rectification was performed with subsequent creation of the linear envelope with a 4th-order recursive low-pass Butterworth filter with a 15 Hz cut-off frequency.

The maximum capacity to generate torque was assessed through the peak torque, which was considered as the maximum torque value from the three MVICs and, at that exact instant, a window of 100 ms was selected (50 ms before and after the peak torque) where the average of the EMG linear envelope was calculated (peak torque EMG). The peak torque EMG gives us an idea about the muscular activation average in the maximum effort and the windowing is necessary to respect the electromechanical delay of the muscle. The peak EMG represents the maximal electrical activity of the muscle and was obtained as the maximum EMG linear envelope curve value from the three MVICs. The quantity of energy produced by the muscular contraction was accessed through the impulse variable and was defined as the integral of the torque curve in the three MVICs. To find the total energy expended by the muscle to produce the contraction, the same was performed in the EMG linear envelope curve to calculate the integral EMG. The neuromechanical efficiency represents the economy of the muscle and was obtained through the ratio between the impulse and the EMG integral.

### 2.5. Statistical Analysis

The statistical analysis was conducted using SPSS Statistics version 27 (IBM Corporation, Armonk, NY, USA), and the significance level was set at α = 0.05. For comparing the different experimental instants and methods, data were normalised as a percentage of the pre-fatigue condition and a multivariate analysis of variance (MANOVA) with repeated measures for the moment factor was used. For comparing the different experimental instants and methods, data were normalised as a percentage of the pre-fatigue condition and a multivariate analysis of variance (MANOVA) with repeated measures for the moment factor was used and not paired between the methods because the muscle groups analysed were differed between the methods. The significance level of the pairwise analysis was corrected according to the Bonferroni method.

## 3. Results

The peak torque, peak EMG, peak torque EMG, integral EMG, impulse and neuromechanical efficiency values assessed during the pre-fatigue, post-fatigue and post-recovery instants are displayed in Table 2. Peak torque was higher in pre- than post-fatigue instants on localised heating, compression and control conditions (15.5, 16.2 and 14.4%, respectively) and between pre-fatigue and post-recovery on electrostimulation (13.6%). Impulse was higher in pre- vs. post-fatigue instants on the localised heating and control groups (18.2 and 15.3%, respectively). No differences were found between the remaining variables and instants. Moreover, we also compared electrostimulation and control conditions, and those comparisons can be seen in Table 3. We choose to standardise the values by this condition, thus the pre-fatigue comparisons are not disclosed.

## 4. Discussion

The aim of the current study was to analyse the acute recovery from a fatigue protocol provided by an intelligent system developed to incorporate three distinct recovery methods (electrostimulation, localised heating and compression). The data indicate that the legging used showed no significant capacity for acute recovery potentiation from a fatigue-inducing protocol in all recovery methods when comparing the post-fatigue and post-recovery instants. This is the first study that has analysed acute recovery using a device that assembles three different recovery methods applied to different muscles. It is known that electrostimulation enhances blood flow, speeding up the removal of muscle metabolites [9] and enhancing recovery by reducing muscle pain through central and peripheral mechanisms [7]. However, the data from the literature are still controversial, showing that electrostimulation is not as effective as expected; however, this might be justified by the heterogeneity of the samples and protocols [10].

Prior research compared electrostimulation to the peroneal nerve behind the knee (1 Hz frequency, 27 mA current and 140 µ.s pulse width) with a passive recovery at 2 and 24 h post-exercise, resulting in improved biochemical markers and perceived muscle soreness, along with an improved recovery of neuromuscular function at 24 h [33]. Complementarily, when analysing if the application of 20 min electrostimulation was effective to recover from an intermittent exercise protocol, we observed a faster restoration of the 30 s all-out performance even if no alterations were found on muscle damage markers [34]. None of these findings were seen in the current study, probably due to the protocol duration and format and our sample characteristics.

A recent review on the outputs of electrostimulation highlighted that no benefit can be obtained regarding power, functional strength measurements or work-related and cardiorespiratory outcomes [10], although data inconsistency was present (probably due to the different experimental protocols used). When electrostimulation, low-intensity exercise and total rest methodologies were compared regarding the recovery of high-intensity training, no differences between them were noticed, even if electrostimulation slightly improved the participants’ subjective perception of recovery immediately after its application [35]. In the current study, electrostimulation had no effect on acute recovery peak torque and the related secondary variables.

Even if we did not observe differences between post-fatigue and post-recovery for localised heating data, lower peak torque values were expected when comparing pre- and post-fatigue for all recovery methods. In fact, isometric strength is generally diminished immediately after post-eccentric exercise and recovery is gradual and prolonged [36]. The underlying mechanisms that justify its application are the elevated muscle tissue temperatures that can upregulate heat shock proteins and other signalling molecules [12], with heat treatment before or after eccentric contractions resulting in an increased recovery of muscular strength, muscle endurance and soreness [13,14]. A recent systematic review on heat and cold therapies for the treatment of delayed-onset muscle soreness showed lower levels of muscle soreness in the experimental group [37]. However, some of the included randomised control trials did not clearly describe the randomisation process or allocation concealment that, together with the heating protocols’ heterogeneity, might have led to bias. In the current study, localised heating was not effective for acute recovery.

The use of external compression is known to affect a few cellular and hemodynamic processes [18], which is due to the mechanical pressure application compressing the tissues underneath [19] reducing the space available for swelling [20]. The use of compression garments in recovery from exercise-induced muscle damage is controversial, with some studies supporting their use [21], other authors reporting no additional benefit [22], and a third party stating that no adverse effects are expected when they are used to recover from damaging exercise [23,24]. Judging by the current study results, compression was not effective for accelerating acute recovery after the fatigue protocol.

The first systematic review on the abovementioned topic revealed that the use of compression garments permits a faster recovery of muscular function, from soreness and of systemic creatine kinase activity, evidencing beneficial effects on the recovery from exercise-induced muscle damage [38]. However, some methodological traits were not considered, particularly the blinding of the patients and unclearness regarding participant sequence generation and allocation concealment. A recent systematic review with meta-analyses found that even when the kind of activity, the body region and the timing of the compression garment application are taken into consideration, wearing compression garments during or after exercise does not appear to assist in the recovery of muscular strength following physical exercise [39].

A recent study aiming to determine if wearing a below-knee compression garment during isokinetic exercise reduced strength loss and the deleterious fatigue effects on joint position sense, showed that the used compression garments failed to reduce those outcomes [40]. In addition, the group that did not use compression garments during exercise had smaller maximum voluntary isometric contraction values and experienced more fatigue. These facts indicate that the used apparel may increase torque values via intramuscular pressure, consequently decreasing the cross section of blood vessels and accelerating the blood flow (as seen before [41,42]) and compensating the effects of fatigue [40].

A simple definition of recovery from exercise-induced muscle damage or from sports exercise is a return to baseline performances; if the athlete is still not at the baseline levels, then they are not fully recovered [43]. Recovery from exercise-induced muscle damage is influenced by many factors such as training level, age, sex, genetics, exercise type and duration [44]. Our sample constituted a very heterogeneous group, i.e., males and females, different resistance exercise experience among the group and different body mass indexes, so the results can be influenced by these factors [45]. Ultimately, other measures such as inflammatory [46] and muscle damage markers [47] could be used at different time points because, although it is individual, the peak delayed-onset muscle soreness usually occurs between 42 and 72 h after exercise [48,49] and a recovery intervention with the legging within those time points would be interesting.

Electromyography was a fundamental part of the current study since it enabled us to analyse muscle activity during maximum voluntary isometric contractions. Being of a non-invasive nature, it is easy to carry out, although it does require expertise in signal acquisition, decomposition, analysis and data interpretation [29], particularly for research purposes [50,51]. Nonetheless, decomposition techniques have had a huge advance in recent decades, enabling the experiential learning of theoretical subjects and annual scientific publications increasing significantly [29]. Isometric contractions can be used for studying muscle recruitment behaviour and motor unit firing, with these maximal voluntary efforts showing a decreasing trend of motor units firing rates [52,53]. Motor units are recruited in an increasing amplitude and decreasing firing rate sequences to reach 70% of the maximum voluntary contraction [29]. However, during a series of fatiguing contractions, the firing rate of motor units in the vastus lateralis muscle first decreased and subsequently increased along with the recruitment of additional motor units [54].

The novelty of the current work relies on the fact that this sports legging is the first to assemble three recovery methods into one single device. However, this study has some limitations, particularly the fact that our sample was relatively small; this may have contributed to the reported lack of differences. Second, our sample comprised tier 1 male and female athletes and a sample such as this is not homogeneous, which probably also affects the results. Lastly, another source of potential bias may have been the impossibility of blinding the participants to the recovery method that they were going to receive, so a placebo or nocebo might have occurred. In the near future, we will test this legging on different tier athletes and compare the results across the spectrum. We will also analyse inflammatory and muscle damage markers, as well as the perceived recovery status.

## 5. Conclusions

Differences in peak torque were expected between pre- and post-fatigue instants, however, no other relevant result was seen in favour of the acute recovery provided by the sports legging. Our data are in line with the most recent available systematic reviews and meta-analyses in the field. It would be interesting to follow our future directions, and it is important to note that this is the first study comparing a device that assembles three recovery methods into a single piece of clothing.

## Figures and Tables

**Table 1:** Different intensities for electrostimulation between the sports legging and the Digitimer^®^.

Participant ID	Legging	Digitimer DS8R
01	20 mA	8.5 mA
02	20 mA	9.5 mA
03	15 mA	5.0 mA
04	10 mA	5.0 mA
05	30 mA	8.0 mA
06	15 mA	4.5mA
07	15 mA	10.0 mA
08	20 mA	4.5 mA

**Table 2:** Comparison between the different instants of analysis and recovery methods.

Measure	Method	Instant	Instant	Mean Differences	*p* Value	d Value	Power
Peak torque	Localised heating	Pre-fatigue	Post-fatigue	15.456	0.001	5.53	1
		Pre-fatigue	Post-recovery	8.247	0.229	1.7	0.93
		Post-fatigue	Post-recovery	−7.209	0.382	2.6	0.99
	Compression	Pre-fatigue	Post-fatigue	16.208	0.001	0.69	0.37
		Pre-fatigue	Post-recovery	8.294	0.181	1.49	0.89
		Post-fatigue	Post-recovery	9.902	0.119	1.55	0.90
	Electrostimulation	Pre-fatigue	Post-fatigue	8.924	0.091	1.99	1
		Pre-fatigue	Post-recovery	13.623	0.014	2.24	0.94
		Post-fatigue	Post-recovery	4.700	0.943	0.73	0.85
	Control	Pre-fatigue	Post-fatigue	14.354	0.001	7.03	0.99
		Pre-fatigue	Post-recovery	4.602	0.926	1.61	0.99
		Post-fatigue	Post-recovery	−4.199	1.000	1.42	0.40
Peak torque EMG	Localised heating	Pre-fatigue	Post-fatigue	3.639	1.000	0.47	0.14
		Pre-fatigue	Post-recovery	−8.955	1.000	0.65	0.23
		Post-fatigue	Post-recovery	−12.595	1.000	0.29	0.08
	Compression	Pre-fatigue	Post-fatigue	−18.604	0.527	1.66	0.87
		Pre-fatigue	Post-recovery	−29.689	0.188	2.1	0.97
		Post-fatigue	Post-recovery	−11.085	1.000	0.73	0.27
	Electrostimulation	Pre-fatigue	Post-fatigue	3.090	1.000	0.5	0.15
		Pre-fatigue	Post-recovery	1.566	1.000	0.5	0.15
		Post-fatigue	Post-recovery	−1.524	1.000	0.71	0.26
	Control	Pre-fatigue	Post-fatigue	−17.651	0.594	5.73	1
		Pre-fatigue	Post-recovery	−21.039	0.542	3.61	0.99
		Post-fatigue	Post-recovery	−3.388	1.000	5.74	1
Impulse	Localised heating	Pre-fatigue	Post-fatigue	18.226	0.014	3.33	0.99
		Pre-fatigue	Post-recovery	11.595	0.604	2.61	0.99
		Post-fatigue	Post-recovery	−6.631	1.000	0.95	0.42
	Compression	Pre-fatigue	Post-fatigue	−3.365	1.000	0.81	0.33
		Pre-fatigue	Post-recovery	9.611	0.957	1.35	0.71
		Post-fatigue	Post-recovery	12.976	0.447	1.59	0.84
	Electrostimulation	Pre-fatigue	Post-fatigue	7.639	0.625	1.71	0.89
		Pre-fatigue	Post-recovery	17.653	0.169	1.91	0.94
		Post-fatigue	Post-recovery	10.014	0.693	0.98	0.45
	Control	Pre-fatigue	Post-fatigue	15.356	0.046	5.71	1
		Pre-fatigue	Post-recovery	3.259	1.000	1	0.46
		Post-fatigue	Post-recovery	−12.098	0.451	2.95	0.99
Peak EMG	Localised heating	Pre-fatigue	Post-fatigue	0.743	1.000	0.9	0.39
		Pre-fatigue	Post-recovery	8.004	1.000	1.05	0.5
		Post-fatigue	Post-recovery	7.260	1.000	0.67	0.24
	Compression	Pre-fatigue	Post-fatigue	−9.637	1.000	1.24	0.64
		Pre-fatigue	Post-recovery	−30.328	0.083	2.14	0.98
		Post-fatigue	Post-recovery	−20.691	0.386	1.28	0.66
	Electrostimulation	Pre-fatigue	Post-fatigue	2.204	1.000	0.86	0.36
		Pre-fatigue	Post-recovery	9.447	1.000	4.55	1
		Post-fatigue	Post-recovery	7.243	1.000	2.31	0.99
	Control	Pre-fatigue	Post-fatigue	−19.930	0.198	2.02	0.96
		Pre-fatigue	Post-recovery	−7.187	1.000	0.82	0.33
		Post-fatigue	Post-recovery	12.743	0.933	0.97	0.44
Integral EMG	Localised heating	Pre-fatigue	Post-fatigue	4.263	1.000	0.56	0.18
		Pre-fatigue	Post-recovery	10.879	0.966	1.26	0.65
		Post-fatigue	Post-recovery	6.616	1.000	0.57	0.18
	Compression	Pre-fatigue	Post-fatigue	−0.901	1.000	0.07	0.05
		Pre-fatigue	Post-recovery	−4.459	1.000	0.63	0.21
		Post-fatigue	Post-recovery	−3.558	1.000	0.25	0.08
	Electrostimulation	Pre-fatigue	Post-fatigue	5.707	1.000	0.94	0.41
		Pre-fatigue	Post-recovery	5.631	1.000	0.71	0.26
		Post-fatigue	Post-recovery	−0.076	1.000	0.007	0.05
	Control	Pre-fatigue	Post-fatigue	−6.836	1.000	0.79	0.31
		Pre-fatigue	Post-recovery	−19.979	0.224	2.79	0.99
		Post-fatigue	Post-recovery	−13.143	0.698	1.18	0.59
Neuromechanical efficiency	Localised heating	Pre-fatigue	Post-fatigue	11.139	1.000	2.32	0.99
		Pre-fatigue	Post-recovery	−13.387	0.855	0.93	0.41
		Post-fatigue	Post-recovery	−24.527	0.201	1.62	0.85
	Compression	Pre-fatigue	Post-fatigue	−20.990	0.290	1.39	0.73
		Pre-fatigue	Post-recovery	8.276	1.000	0.93	0.41
		Post-fatigue	Post-recovery	29.266	0.127	1.67	0.87
	Electrostimulation	Pre-fatigue	Post-fatigue	−0.303	1.000	0.86	0.36
		Pre-fatigue	Post-recovery	13.828	0.810	3.18	0.99
		Post-fatigue	Post-recovery	14.130	0.843	2.35	0.99
	Control	Pre-fatigue	Post-fatigue	14.714	0.624	0.7	0.05
		Pre-fatigue	Post-recovery	17.474	0.498	6.86	1
		Post-fatigue	Post-recovery	2.760	1.000	2.63	0.99

**Table 3:** Comparison between the electrostimulation and control condition.

Measure	Instant	Mean Differences	*p* Value	d	Power
Peak torque	Pre-fatigue	-	-	-	-
	Post-fatigue	2.299	1.000	0.29	0.08
	Post-recovery	−6.085	1.000	0.76	0.29
Peak torque EMG	Pre-fatigue	-	-	-	-
	Post-fatigue	−21.597	1.000	2.17	0.98
	Post-recovery	−15.895	1.000	1.97	0.96
Impulse	Pre-fatigue	-	-	-	-
	Post-fatigue	7.718	1.000	0.97	0.44
	Post-recovery	−14.393	1.000	1.8	0.92
Peak EMG	Pre-fatigue	-	-	-	-
	Post-fatigue	−22.134	0.863	2.77	0.99
	Post-recovery	−16.634	1.000	2.08	0.97
Integral EMG	Pre-fatigue	-	-	-	-
	Post-fatigue	−12.543	1.000	1.57	0.83
	Post-recovery	−25.610	0.627	3.2	0.99
Neuromechanical efficiency	Pre-fatigue	-	-	-	-
	Post-fatigue	15.017	1.000	1.87	0.93
	Post-recovery	3.647	1.000	0.46	0.14

## Data Availability

To request data, contact us by email up201807261@fade.up.pt.
